# The Yeast *Saccharomyces cerevisiae* as a Model for Understanding RAS Proteins and Their Role in Human Tumorigenesis

**DOI:** 10.3390/cells7020014

**Published:** 2018-02-19

**Authors:** Giulia Cazzanelli, Flávia Pereira, Sara Alves, Rita Francisco, Luísa Azevedo, Patrícia Dias Carvalho, Ana Almeida, Manuela Côrte-Real, Maria José Oliveira, Cândida Lucas, Maria João Sousa, Ana Preto

**Affiliations:** 1 CBMA—Centre of Molecular and Environmental Biology, Department of Biology, University of Minho, Campus de Gualtar, 4710-057 Braga, Portugal; giulia.cazzanelli88@gmail.com (G.C.); flaviabrandao.fcbp@gmail.com (F.P.); sara.csa@gmail.com (S.A.); rita.francisco.28@gmail.com (R.F.); pat.dcarvalho@gmail.com (P.D.C.); anafmalmeida@gmail.com (A.A.); mcortereal@bio.uminho.pt (M.C.R.); clucas@bio.uminho.pt (C.L.); 2 Instituto de Investigação e Inovação em Saúde, Universidade do Porto, Rua Alfredo Allen 208, 4200-135 Porto, Portugal; mariajo@ineb.up.pt (M.J.O.); 3 New Therapies Group, INEB-Institute for Biomedical Engineering, 4200-135 Porto, Portugal; 4 IPATIMUP-Institute of Molecular Pathology and Immunology, University of Porto, Rua Júlio Amaral de Carvalho 45, 4200-135 Porto, Portugal; lazevedo@ipatimup.pt (L.A.); 5 Department of Biology, Faculty of Sciences, University of Porto, Rua do Campo Alegre S/N, 4169-007 Porto, Portugal

**Keywords:** RAS proteins, *S. cerevisiae*, model, homologues, colorectal cancer, autophagy, KRAS

## Abstract

The exploitation of the yeast *Saccharomyces cerevisiae* as a biological model for the investigation of complex molecular processes conserved in multicellular organisms, such as humans, has allowed fundamental biological discoveries. When comparing yeast and human proteins, it is clear that both amino acid sequences and protein functions are often very well conserved. One example of the high degree of conservation between human and yeast proteins is highlighted by the members of the RAS family. Indeed, the study of the signaling pathways regulated by RAS in yeast cells led to the discovery of properties that were often found interchangeable with RAS proto-oncogenes in human pathways, and vice versa. In this work, we performed an updated critical literature review on human and yeast RAS pathways, specifically highlighting the similarities and differences between them. Moreover, we emphasized the contribution of studying yeast RAS pathways for the understanding of human RAS and how this model organism can contribute to unveil the roles of RAS oncoproteins in the regulation of mechanisms important in the tumorigenic process, like autophagy.

## 1. Introduction

The exploitation of eukaryotic organisms as biological models has been fundamental for biological discoveries up to the present day. Among those models, a special position is reserved for the yeast *Saccharomyces cerevisiae*, which continues to be extremely useful for the investigation of basic cellular processes conserved in complex multicellular organisms such as humans, profiting from the availability of a larger set of resourceful techniques [1,2,3]. Almost thirty years ago, the power of yeast as a model organism was already clear, due to “the facility with which the relation between gene structure and protein function can be established” [4]. 

Importantly, in 1996 *S. cerevisiae* was the first eukaryote to have its complete genome sequenced and published [5] and continuous updates have been made since [6]. A few years later, a set of yeast strains with deletions of most of its annotated open reading frames (ORF) was made available [7,8] and currently, relatively simple methods for introducing gene mutations are well established, allowing the discovery of the biochemical function of the analyzed gene and the outcomes of the gene loss [1]. Supported by these resources, the research on *S. cerevisiae* had important repercussion for unveiling the role of many different proteins in the biology of human cells [1,3]. This was possible because of the high degree of conservation of many of the yeast and human proteins, at the level of both amino acid sequence and function. 

One such example of a high degree of conservation is the case of the members of the RAS family of proteins [9,10,11]. *RAS* genes are the founding members and prototypes of the RAS superfamily of small guanosine triphosphatases (GTPases). The “classical” mammalian RAS proto-oncogenes (HRAS, KRAS and NRAS) are the most extensively studied among all the RAS superfamily members because of their direct involvement in tumorigenesis. The members of RAS are involved in cell proliferation, gene expression, differentiation, migration/invasion, autophagy and apoptosis [12,13,14,15,16,17]. The interest in RAS began in the 1960s with the discovery of Harvey and Kirsten rat sarcoma retroviruses, which were identified as viral genes transduced from the rodent genome and responsible for causing tumors in mice. These genes were respectively termed v-HRAS and v-KRAS [18,19]. Nevertheless, it was only in 1982, with the identification of activated mutant forms of these genes in human cancer cell lines, that intensive biochemical, biological and structural studies of RAS began [20]. In addition to the previously described RAS isoforms, a third isoform was identified in 1983 and named neuroblastoma (N-) RAS [21]. In the same period, the two *RAS* yeast homologues, *RAS1* and *RAS2*, were identified based on DNA sequence similarity with KRAS and HRAS [9]. The yeast Ras proteins were isolated [22] and their nucleotide sequences were determined [10,11]. The importance of the study of yeast RAS proteins in elucidating mammalian RAS regulation and roles was immediately clear [23,24,25,26,27]. Since then, the studies of the RAS pathway in humans has led to the discovery of properties that were often found to be interchangeable with the yeast RAS pathway, and vice versa, due to the high degree of similarity among their protein members and upstream regulators. However, it is important to stress that human and yeast proteins are not identical, and likewise, the pathways they control also differ. 

In this work, we performed an updated critical literature review on human and yeast RAS pathways, specifically highlighting the similarities and differences between them. Moreover, we emphasized the contribution of studying yeast RAS pathway for the understanding of human RAS oncoproteins and how this model organism can contribute to the unveiling of their role in the regulation of mechanisms important in the tumorigenic process, like autophagy.

## 2. *S. cerevisiae* as a Model Organism for Studying Human Proteins and Molecular Mechanisms Underlying Associated Diseases

Current understanding of basic aspects of different cell processes, such as cell cycle, DNA replication, vesicular trafficking, aging and cell death has received a major contribution from studies on *S. cerevisiae* [1], supporting the use of this organism as a powerful experimental system. Different factors contribute to the success of yeast as a model organism. First of all, *S. cerevisiae* is a eukaryote, so it shares the cellular structure and organization of higher eukaryotic cells, such as mammalian cells. Secondly, when comparing yeast and other organisms, it is clear that both amino acid sequences and protein functions are conserved. Thirdly, as mentioned above, a broad range of specific molecular tools and resources are available in yeast. Indeed, besides the sequenced genome [5] and a set of yeast ORF deletion strains [7,8], other collections with genome-wide coverage are available, such as a collection of GFP-fused chimera proteins that helps localize endogenous yeast proteins [28,29]. Moreover, the Saccharomyces Genome Database (http://www.yeastgenome.org/), which gives detailed and updated information about every yeast gene, is available. All these resources have made it possible to uncover the role of almost 85% of the 5800 protein-coding genes of *S. cerevisiae*, with important repercussions for the biology of other organisms, including humans. 

**Figure 1:**
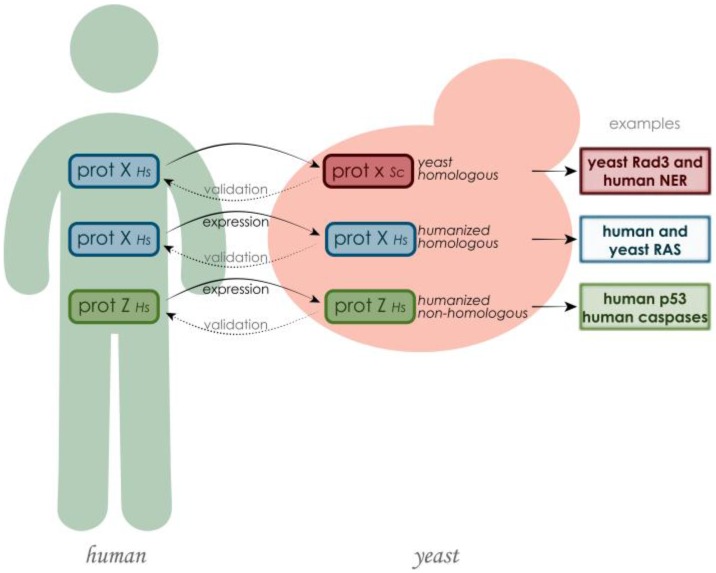
The study of human proteins putatively involved in disease using *S. cerevisiae* as model can be summarized in three main methodologies. If the human protein has a yeast counterpart, the yeast protein can be studied in its environment and its function can be compared with the one in human cells, or the human gene can be cloned and expressed in yeast, in order to be studied in a neutral environment. Also human proteins that do not have a yeast orthologue can be cloned in yeast, especially with the purpose of finding their inhibitors or activators. In every case, the discoveries made in yeast need further validation in human cells.

Approximately 17% of yeast genes are members of orthologous gene families associated with human diseases [30] and, conversely, 30% of known genes involved in human diseases have yeast orthologues and can substitute for yeast gene function [31]. The fact that many of the protein functions discovered in yeast can then be translated to higher eukaryotes evidences the relevance of using this model in the study of proteins involved in human disorders. At first glance, it might seem that yeast proteins have little to do with processes involved in human diseases. However, some of these processes can be better analyzed in the simpler environment of yeast cells, like in the case of the aggregations of misfolded proteins implicated in neurodegenerative disorders, such as Parkinson’s, Huntington’s and Alzheimer’s diseases [2,3,32,33]. Besides studying yeast protein functions and then translating them to higher eukaryotes, a different approach can be considered to directly understand the role of human proteins: the creation of humanized yeast by heterologously expressing human proteins [34] (Figure 1). This strategy is particularly helpful in the discovery of human protein functions associated with a disease in a “neutral” environment, devoid of the different layers of complexity that were acquired during evolution. It is also useful for determining the outcome of protein mutations [1]. The expression of human proteins in yeast can also be instrumental in the discovery of chemical or protein inhibitors of their activity in high-throughput screenings. These assays are based on the fact that human proteins expressed heterologously may cause growth defects in yeast, which can be suppressed by chemical compounds or by the expression of a second human protein [3]. Yeasts have also been used to express human genes that do not have an endogenous functional counterpart. This approach has been successfully exploited in the study of neurological disorders such as Huntington’s, Alzheimer’s and Parkinson’s diseases [32,33,35,36,37,38].

Importantly, yeast has been applied in the study of proteins relevant for tumorigenesis [39,40] and in the discovery and testing of anticancer agents [41,42,43,44]. For example, yeast deletion mutant collections have been used to identify genes involved in UV sensitivity, and the correspondent human orthologues putatively associated with cancer development [45], and to screen for drug effects, for instance to test sensitivity and/or resistance to the common anticancer drug bleomycin [46]. Several cellular processes important in cancer onset and progression, such apoptosis and cell growth, have also been analyzed in yeast. The heterologous expression of caspases in yeast greatly helped to uncover their mechanism of activation [47]. Importantly, high expression level of caspases in yeast caused severe growth defects, and this phenotype facilitated the identification of caspase natural inhibitors, such as IAPs and p35 [47,48], as well as chemical inhibitors and activators [49]. Bcl-2 family members have also been studied in yeast. A Bcl-2 family pro-apoptotic member, Bax, is capable to induce cell death in yeast. Screening for its inhibitors led to the discovery of Bax inhibitor 1, Bar1, HMGB1, bifunctional apoptosis regulator and Calnexin orthologue Cnx1 [50,51,52,53,54], and other inhibitors belonging to the Bcl-2 family, such as Bcl-2 and Bcl-X_L_ [55]. Studies on p53, one of the most important proteins in cancer development, have also been addressed in yeast. p53 is mutated in approximately 50% of human cancers and, when not mutated, often other proteins involved in p53-mediated pathways are non-functional [56]. There are no orthologues of p53 in yeast, but this protein can maintain most of its activity as transcription factor in *S. cerevisiae* [57,58,59]. Aside from the conserved function, p53 can cause a mild decrease of yeast growth [60,61]. All these factors have been extensively exploited to better analyze the function and regulation of p53. For example, p53 regulation by redox level and thioredoxin reductase were first discovered in yeast and then confirmed in mammals [62,63]. Another study revealed the conservation in yeast of functional transcription-dependent and -independent p53 apoptotic mechanisms [64]. Also the importance of p53 mutations for pathogenesis has been analyzed in yeast, facilitated by the amenability of yeast high-throughput assays. Indeed, all the mutations of p53 representing amino acid substitutions were expressed in yeast and tested for various aspects, including the ability to activate proteins involved in cell cycle and apoptosis [65,66,67].

### S. cerevisiae as a Model Organism for Studying Human RAS Proteins

Human RAS proteins have a role in tumorigenesis and are highly conserved in yeast [9,10,11]. Indeed, though they do not activate the same downstream pathways, the upstream regulating events and the resulting effects are often very similar. Therefore, the usage of *S. cerevisiae* as model for dissecting the role of these oncoproteins and the underlying molecular mechanisms was not only possible, but was also extremely useful.

Historically, the study of yeast RAS pathway has brought great insights in the understanding of mammalian RAS pathway. First of all, yeast was very useful for understanding the post-translational process necessary for RAS proteins to translocate to the plasma membrane [68]. In particular, the usage of a *S. cerevisiae* mutant, *dpr1Δ*, made clear that acylation could not be the first translational modification in the processing of RAS proteins [69]. Moreover, the first RAS effector, adenylate cyclase, and the first guanine-nucleotide exchange factor (GEF), Cdc25, were identified in yeast [70,71,72]. These discoveries, especially the one of Cdc25, helped to uncover similar proteins in other organisms, including humans, based on homology [73,74,75]. In addition to GEF proteins, yeast has been useful to deepen the understanding of the other class of RAS regulators, GTPase activating proteins (GAPs), IRA in yeast. Indeed, the functions of GAP-coding *NF1* gene, whose mutations are responsible for neurofibromatosis type 1, were clarified thanks to the similarity with *IRA* genes of *S. cerevisiae* [76]. Importantly, expression of *NF1* in yeast can suppress the phenotypes caused by deletion of *IRA* genes, such as heat shock sensitivity [77], proving that mammalian and yeast GAPs are interchangeable and highlighting the similarity between yeast and mammalian RAS proteins activation. 

## 3. Human and Yeast RAS Proteins: Similarities and Differences

The human RAS family includes three genes: *HRAS, NRAS*, and *KRAS*. These three loci encode four different protein isoforms: HRAS, NRAS, KRAS4A, and KRAS4B. The two KRAS isoforms differ due to the alternative splicing of exon 4 in the KRAS locus. KRAS4A is expressed at low levels, whereas KRAS4B (hereafter referred to as KRAS) is ubiquitously expressed and accounts for 90–99% of all KRAS mRNA [12,78,79,80]. All isoforms are similar (~85%) in their primary amino acid sequence in the G-domain, which is responsible for GTP/GDP binding, whereas the major differences are concentrated in the hypervariable region (HVR) at the C terminus, which is particularly important for post translational modifications (PTMs) and consequent intracellular targeting [81]. Despite their high conservation at the amino acid sequence level, their functions differ significantly and they do not display redundant functionality. The specific roles of RAS proteins may be explained by various factors, such as cellular context, differential interaction with effectors, compartmentalized signaling and PTMs [82]. While HRAS, KRAS4A and NRAS have been shown to be dispensable for normal development in mice, KRAS4B knockout was proven to be embryonically lethal [12]. 

*S. cerevisiae* expresses two proteins homologous to human RAS, Ras1 and Ras2. *RAS1* and *RAS2* genes are located in chromosome XV and XIV, respectively [25,83], and encode two highly similar proteins of 36 and 40 kDa [22,84]. Ras1 and Ras2 have the same function, but different expression regulation [85,86]. Initially, they were thought to have different roles in the cell, because Ras1 could not complement growth defects caused by the deletion of *RAS2*, such as reduced growth on non-fermentable carbon sources and low level of intracellular cAMP [25,87,88], and *RAS1* mutants did not present any clear phenotype. However, these effects are now explained by the different regulation of the mRNA production and of protein translation of *RAS1*. Indeed, the level of *RAS1* mRNA and protein synthesis are reduced as cells approach mid-logarithmic phase and when cells are grown on non-fermentable carbon sources [85], while *RAS2* mRNA levels are high during growth phases [86] and on both fermentable and non-fermentable carbon sources [85]. The final confirmation that the only difference between *RAS1* and *RAS2* lies in differential expression was obtained by expressing *RAS1* under the constitutive *ADH1* promoter. In this case, *RAS1* was able to fully suppress the phenotype of *Δras2* and the hyperactive mutant of *RAS1* showed the same effects as the constitutively expressed *RAS2* [89]. 

**Figure 2:**
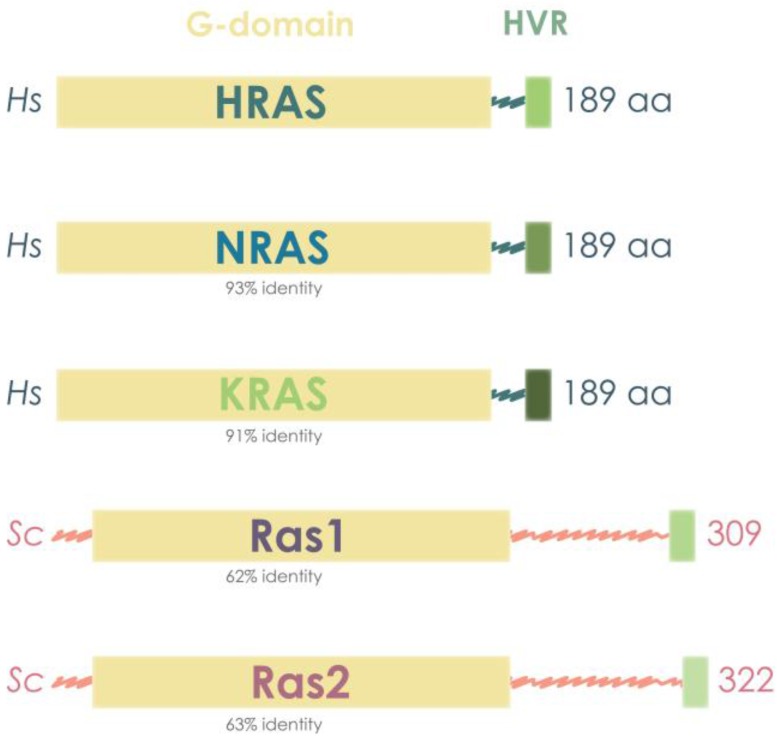
Yeast and human RAS proteins present a highly conserved G domain, which is responsible for GTP/GDP binding. Human RAS proteins are also highly similar among them, HRAS showing 91% of amino acid identity in the G-domain to NRAS and 93% to KRAS. Yeast Ras proteins are bigger, having more than 100 extra amino acids, but still present around 60% amino acid identity in the G-domain to human RAS proteins.

As observed in comparisons within human RAS isoforms and within yeast RAS isoforms, the similarity between human and yeast RAS also resides in the functional G-domain of 180 amino acids, whereas the region of divergence is located at the C-terminal, corresponding to the HVR [9,10,11]. In addition, the yeast RAS molecular weights are higher than those of human RAS, with the size of the proteins being approximately 40 kDa, in contrast with mammalian RAS, which accounts for 21 kDa [22,90] (Figure 2).

### 3.1. Human and Yeast RAS Sequence and Structure 

**Figure 3:**
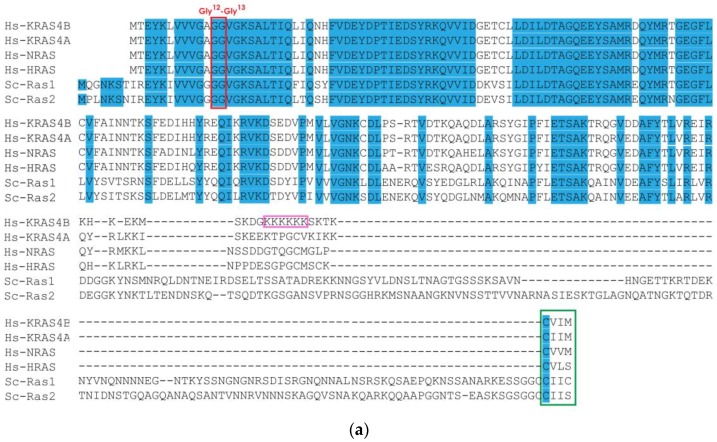

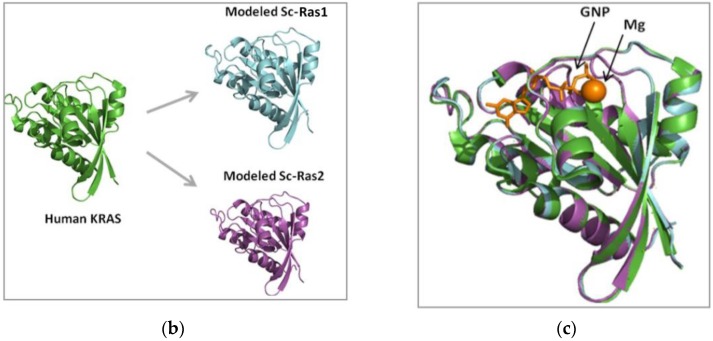
Comparison of RAS proteins between human (Hs) and yeast (Sc) species. (**a**) Alignment of human KRAS, NRAS and HRAS with yeast Ras1 and Ras2. Identical residues at homologous positions are shown in blue. The Gly12 and Gly13 sites are highlighted in red. The CAAX box is highlighted in green. The lysine repeats of KRAS4A are highlighted in pink. Human sequences were obtained from Ensembl (KRAS4B: ENSP00000308495, KRAS4A: ENSP00000256078, NRAS: ENSP00000358548 and HRAS: ENSP00000309845) and yeast sequences from the *Saccharomyces* Genome Database (RAS1: YOR101W and RAS2: YNL098C). Sequences were aligned in Geneious 5.5.8 [94], using Muscle [95]. (**b**) Models of *S. cerevisiae* Ras1 (blue) and Ras2 (purple) using human KRAS structure (3GFT) as a template (green). (**c**) The three structures are shown superimposed, revealing the fold similarity. GDP and Mg sites are shown in orange.

We performed a comparative analysis of yeast and human RAS amino acid sequences and confirmed the high similarity in sequence and structure between human and yeast RAS proteins (Figure 3). Specifically, the N-terminal region is strongly conserved, especially in the first half of the sequence. This reflects the important functional constraints involved in the recognition of guanine nucleotide and phosphate, for which the N-terminal is responsible (Figure 3a). In this segment, 58% of residues are identical between any of the yeast RAS proteins and any of the mammalian RAS sequences. As expected, G12 and G13 residues, which are frequently found mutated in cancer, are among those invariant positions (Figure 3a). The human KRAS structure (PDB ID 3GFT) was used to infer the structure of the yeast RAS (Figure 3b) through homology modeling, as previously documented [91,92]. This comparative analysis evidences the similarity between human KRAS and both yeast RAS proteins (Figure 3c) and suggests that the differences in amino acid sequence would still result in a similar fold in humans and yeast proteins (Figure 3c). The main difference between the three mammalian RAS isoforms (KRAS, HRAS and NRAS) and yeast homologues lies at the C-terminal domain, in the HVR, which is critical to membrane localization and function (Figure 3a). Interestingly, yeast RAS proteins contain an extra C-terminal portion not present in RAS protein from other organisms (Figure 3a). The extended C-terminal accounts for 120 aa in the case of Ras1 and 131 aa for Ras2, making yeast RAS HVR around 140 aa [89]. It has not been established yet if this part can form a secondary structure, however its function has been identified. The extended C-terminal was reported to serve as a negative regulatory domain for RAS, promoting its interaction with GDP and therefore the permanence in the inactive status [93]. Accordingly, it was shown that yeast cells expressing truncated RAS proteins lacking the C-terminal domain (or mammalian RAS proteins, without the extra 120 aa) do not require a functional GEF to be viable, while they do when expressing normal RAS proteins [89].

### 3.2. RAS Mechanism of Action and Regulators 

Since all RAS proteins share a conserved G-domain, which is the functional domain that binds to GTP/GDP, they all present a common mechanism of action. All RAS proteins work substantially as binary molecules switching between an inactive state, in which they are bound to GDP, and an active state, in which they are bound to GTP. Our understanding of the molecular mechanism underlying the activation of RAS proteins has been greatly facilitated by the similarity between human and yeast RAS proteins. Specifically, the relevance of a glutamine in position 61 for the intrinsic hydrolysis activity of human RAS [96,97] was better understood and confirmed by the comparison with yeast. Indeed, the substitution of the glutamine with a leucine in both human and yeast, position 61 and 68, respectively, reduced the GTPase activity of human and yeast RAS proteins [98]. The activation of RAS proteins leads to the subsequent activation of a signaling cascade, which differs depending on the organism and the specific RAS protein [99,100]. Even though the proteins that interact with RAS for the activation of downstream signaling are different in different organisms and for different RAS isoforms in the same organism, most of them share a conserved domain to interact with RAS proteins, the RAS-binding domain (RBD) or RAS association (RA) domain [100]. Many RAS effectors have been identified by screening cDNA libraries for the presence of RA domain [101,102,103]. The conserved working mechanism of RAS proteins relies on their conserved sequence, in particular on the presence of a set (1 to 5) of G box GDP/GTP-binding motif elements beginning at the N-terminal domain, which together form a G-domain of approximately 20 kDa [104]. Particularly relevant among the G box motifs are switch I and switch II, which regulate the conformational changes between GTP- and GDP-bound RAS proteins [105,106]. The conformations of active and inactive state show pronounced changes corresponding to the regions of switch I and II, and these small variations lead to different affinities of active or inactive RAS proteins toward RAS regulators and effectors [107,108,109]. 

**Figure 4:**
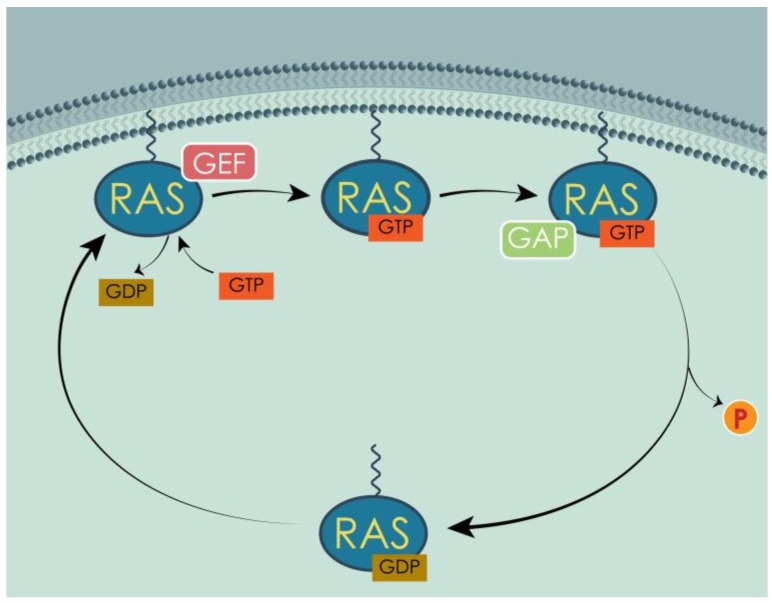
Inactive RAS proteins bound to GDP are localized in the cytoplasm. GEFs catalyze the liberation of GDP from the binding site, allowing GTP, more abundant in the cell, to bind instead. GTP-bound RAS are translocated to the membrane and activated. GAPs enhance the endogenous GTPase activity, hydrolyzing GTP to GDP. RAS is inactive again and goes back to the cytoplasm, where the cycle can begin again, upon proper stimulus.

RAS protein switching between GTP and GDP binding is regulated by two classes of proteins: GAPs and GEFs. GAPs enhance the intrinsically low GTPase activity of RAS proteins, up to 300-fold acceleration [110], in order to reinstate the GDP-bound form of RAS proteins [111], whereas GEFs promote the formation of GTP-bound form, by triggering the dissociation of GDP from RAS proteins, allowing more abundant GTP to bind in its place [112] (Figure 4). As mentioned above, the similarity between human and yeast RAS was a great contribution to the discovery of GEF and their relevance for the proper switch between active and inactive RAS. Indeed, the first GEF, Cdc25, was identified in *S. cerevisiae* [70,71,72]. Based on the homology with Cdc25 and the *Drosophila* GEF, mammalian GEFs were also discovered and identified as RAS switch regulators [73,74,75,113]. The similarities between RAS regulators, in addition to the ones between RAS proteins themselves, contributed to clarifying some aspects of the RAS mechanism. For example, comparing the sequences of various GAPs, including Ira1 and Ira2 from *S. cerevisiae* and human p120GAP, was very important in understanding which regions of the protein were fundamental for the interaction with RAS [96]. Importantly, to reinforce the similarity between human and yeast RAS regulators, yeast Cdc25 can act as GEF for human RAS [114] and mammalian GAPs can enhance the GTPase activity of yeast RAS proteins [77,115], making GEFs and GAPs interchangeable between human and yeast. 

### 3.3. Post-Translational Modifications of Human and Yeast RAS

The activity of both human and yeast RAS proteins is not regulated only by their GTP/GDP binding state, but also by PTMs, which determine RAS sub-cellular localization. Indeed, the localization of RAS proteins at the plasma membrane or on endomembranes establishes the class of effectors and regulators available, determining in turn the downstream signaling [99,100]. As happened in the case of the protein regulators of RAS, GAPs and GEFs, yeast had a pivotal role in the study of the PTMs of RAS. In particular, the study in yeast led to the understanding that RAS processing in the CAAX box was necessary for its translocation to the membrane and, therefore, for its proper activity [69,90,116]. Yeast also contributed to the determination of the sequence of PTMs necessary for RAS processing [117,118]. The discoveries made in yeast were soon translated for human RAS, when it was observed that a human RAS protein expressed in yeast underwent the same PTMs in order to function [68].

RAS proteins are synthesized as cytoplasmic proteins [119] and then undergo different PTMs that fully activate and target them to the inner leaflet of the plasma membrane [100]. The C-terminal domain is necessary for this sub-cellular localization, because it contains the CAAX (C = cysteine, A = aliphatic amino acid, X = terminal amino acid) motif indispensable for membrane targeting [99,100,120]. The cysteine in the CAAX motif is the target of farnesyltransferase, which catalyzes the addition of a farnesyl isoprenoid [99,100,121,122,123]. It has been proved experimentally that the addition of a geranylgeranyl isoprenoid (geranylgeranylation) to the CAAX motif can substitute the farnesylation [124]. However, this PTM has only been observed when farnesyltransferase was blocked by specific inhibitors [124,125,126]. This modification is the first step of RAS processing, followed by other modifications, such as palmitoylation, driven by a second signal contained in the HVR [100]. These two steps are the minimum signal required for transit to and tenure at a membrane, either plasmatic or of internal organelles. This specific differentiation, plasma or endo-membranes, depends exactly on the second step of RAS proteins processing, which is often different for distinct RAS isoforms. 

### 3.4. Differential Localization of Human and Yeast RAS

The localization of RAS proteins in either biological system has always been a matter of debate, with interesting results from both mammalians and yeasts. In both organisms, RAS proteins can exhibit intracellular localizations, but are almost always associated with membranes. Notably, the specific site in which they reside dictates their function and effects, through the interaction with specific partners [99,100].

**Figure 5:**
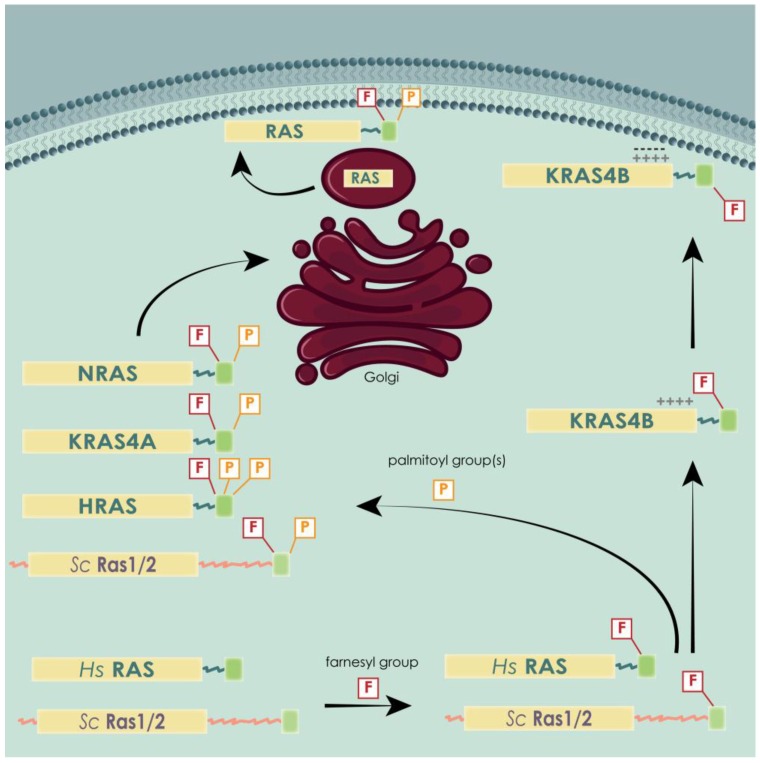
All RAS isoforms undergo a first farnesylation, followed by single palmitoylation in the case of KRAS4A and NRAS, double palmitoylation in the case of HRAS, and no palmitoylation in the case of KRAS4B. This splicing variant is retained in the inner leaflet of the plasma membrane by electrostatic interaction between the positively charged lysines and the negatively charged phospholipids. The three palmitoylated isoforms first pass through the Golgi and are then transported to the membrane via vesicular trafficking. Yeast RAS proteins behave very similar to the palmitoylated human isoforms, also being palmitoylated.

Localization of RAS proteins in mammals varies throughout their lifetime. Once in the active form, i.e. farnesylated, RAS proteins organize themselves in pools situated in the Golgi, the endocytic compartments and the endoplasmic reticulum (ER) [127,128,129]. From that step forward, the processing of the different RAS isoforms differs. HRAS is palmitoylated on C181 and C184, NRAS is palmitoylated only on C181 and KRAS4B is not palmitoylated at all [122,130,131]. KRAS4B contains a polybasic region constituted by a stretch of lysines that enables an electrostatic interaction with the negatively charged plasma membrane phospholipids [122,124,130,132,133] and excludes it from a Golgi-dependent trafficking pathway [127]. Once the CAAX processing is complete, RAS isoforms follow different routes. Palmitoylated isoforms visit the Golgi, where they are acylated and thereby trapped in its membranes, from where they traffic, via vesicular transport, to the plasma membrane [134]. KRAS4A is believed to follow this pathway, as this splice variant does not have the polybasic domain of KRAS4B and it is palmitoylated at C180 [131]. In contrast, KRAS4B cannot be trapped in the Golgi and is directly routed from the endoplasmic reticulum to the plasma membrane by a still poorly understood delivery system that could involve cytosolic chaperones [134] (Figure 5).

**Figure 6:**
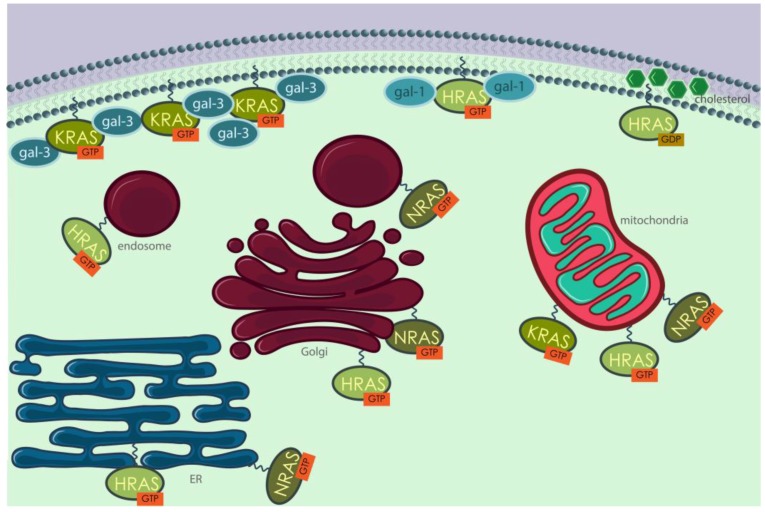
Human RAS proteins are localized in different cellular compartments, almost always associated with membranes. KRAS and HRAS are found in the inner leaflet of the plasma membrane, associated with galectin-3 [151,152] and galectin-1 [153], respectively. Inactive HRAS is very often located in cholesterol-rich structures called caveolae. RAS proteins can also signal from the membranes of internal organelles, such as ER, Golgi, mitochondria and the endosomal system.

In addition to different PTMs, RAS isoforms also display different final localizations on the membrane. For example, Prior et al. [135] demonstrated, through electron and confocal microscopy, that HRAS in its inactive state is predominantly localized at cholesterol-rich domains inside or outside caveolin-rich domains called caveolae, whereas upon activation it delocalizes to disordered regions of the plasma membrane. As for KRAS, it was described to be located mostly outside caveolae and lipid rafts and preferably in electron-dense regions [135]. Once localized at a membrane, either plasmatic or of an internal organelle, RAS isoforms are not dispersed randomly, but are grouped in organized subdomains, as firstly suggested by Roy et al. [136]. Clustering of RAS proteins at the plasma membrane seems to be related with galectins, namely galectin 1 and 3 [137] and cholesterol content [129,136,138,139]. For many years, plasma membrane localization was considered the main platform from which RAS proteins activated their effectors [140]. However, increasing evidences show that RAS proteins are still capable of activating their signaling pathways when located on endomembranes [127,128,140,141]. The presence in the endosomal system of adaptor proteins that are required to activate RAS [142,143] is a strong indicator that RAS proteins could localize in internal cell compartments and signal from there [142,144]. As further evidence, it was shown that ubiquitination of HRAS and NRAS alters their sub-cellular localization to endosomes [145]. KRAS translocates from the plasma membrane to the early and late endosomes through a clathrin-dependent pathway and independently of CaM and protein kinase C phosphorylation [146]. From late endosomes (LEs), KRAS is eventually targeted to lysosomes, as indicated by confocal microscope images showing that GFP-KRAS co-localized, to some extent, with both LAMP1 and LAMP2 lysosomal markers. Fluorescent probes revealed that KRAS was active on LEs, and recruited RAF to initiate a MAP kinase signaling cascade [146]. A study from Chiu et al. [140] showed that HRAS and NRAS engaged their RBD in Golgi and ER after stimulation with common mitogens, such as epidermal growth factor (EGF) and insulin, and from there they were able to activate different signaling pathways with different efficiency. KRAS was found to relocate from the plasma membrane to ER and Golgi after being phosphorylated by protein kinase C on serine 181 [147] and to move along the endocytic compartment maintaining the ability to activate MAPK (mitogen-activated protein kinase) upon EGF stimulation [146]. Association with mitochondria has also been reported [148] (Figure 6). 

In conclusion, RAS post-translational modifications address different RAS isoforms to different localizations, specifically different intracellular organelles, and determine the relative proportion of each isoform in each location. Typically, the relative contribution on endomembranes is NRAS > HRAS and KRAS4A > KRAS4B [149,150], with KRAS4B being more often associated with plasma membranes, whereas NRAS is more frequently found on endomembranes [149]. 

Yeast RAS proteins also localize in different cell compartments in order to activate different effectors and thereby to control different processes [154]. Indeed, fluorescent tagging of yeast RAS proteins and their partners, including the GAP Ira proteins and the GEF Cdc25, showed that most of these molecules are actually localized on endomembranes [154]. In particular, Ras2 activates adenylyl cyclase on the plasma membrane and is engaged to the ER by Ras inhibitor 1 (Eri1) [155]. It has also been reported to localize at mitochondrial membranes, together with Ira proteins [154]. More recent studies go further, showing that the localization of active RAS is dependent on PKA activity [156,157].

In summary, the compartmentalized RAS activities have now been validated in many cell types across species. Therefore, the exclusively plasma membrane localization of RAS proteins is evidently an oversimplification [158]. 

### 3.5. Human and Yeast RAS Downstream Signaling Pathway

As aforementioned, many studies have highlighted the great similarities between human and yeast RAS proteins at the level of sequence, structure, mechanism of action, PTMs and differential localization. On the other hand, the most evident differences between the intracellular activities of the RAS proteins in the two organisms relate to RAS effectors and their downstream signaling cascades. 

**Figure 7:**
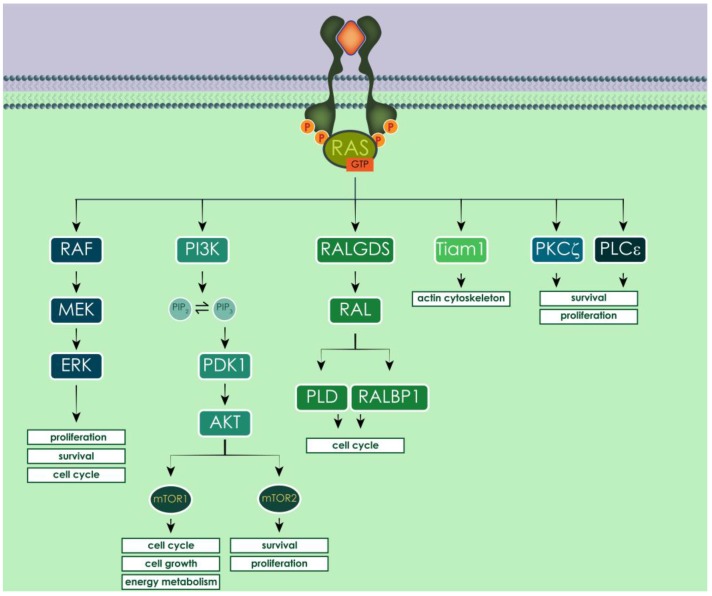
Activated human RAS proteins can activate multiple effectors, having different impact on cell fate. Among the most studied, activated RAF triggers pro-growth signaling through the MAP kinases cascade; PI3K leads to AKT activation and RALGDS stimulated cell cycle progression.

Human RAS proteins work as transducers of pro-survival signals from the extracellular space to the intracellular compartment, through different tyrosine kinases receptors (TKRs), among which the most studied is the epidermal growth factor receptor (EGFR). The dimerization of the receptors upon ligand binding triggers a conformational change that activates the catalytic tyrosine kinase domain, enabling the autophosphorylation of the intracellular carboxyl-terminal domain and therefore its activation [159,160,161]. The phosphorylated intracellular domain of TKR recruits GEFs, which activate RAS, enabling it to signal downstream [162]. Activated RAS proteins can bind and activate at least 20 different effectors, among which the best known and characterized are RAF (rapidly-accelerated fibrosarcoma) kinases, phosphatidylinositol 3-kinase (PI3K) and RAL guanine nucleotide dissociation stimulator (RALGDS). Other effectors of RAS proteins are RIN1, T lymphoma invasion and metastasis-inducing 1 (Tiam 1), Af6, Nore1, PLCε and PKCζ [131,163,164,165] (Figure 7).

Activated RAF kinases phosphorylate and activate MEK (MAPK/ERK kinase), leading to the activation of ERK (extracellular signal-regulated kinase, also called MAPK). ERK regulates several transcriptional factors that influence cell cycle progression, proliferation and survival (e.g., autophagy) [21]. In the other pathway, activated PI3K catalyzes the production of PIP_3_ (phosphatidylinositol-3,4-triphosphate) by phosphorylating PIP_2_ (phosphatidylinositol-4,5-diphosphate), a process reversed by the phosphatase PTEN (phosphatase and tensin homolog deleted in chromosome ten). PIP_3_ activates phosphatidylinositol dependent kinase 1 (PDK1), which recruits AKT to the plasma membrane and activates it [166]. The main downstream effector of activated AKT is mTOR (mammalian target of rapamycin), which is an atypical serine/threonine kinase that can form two distinct complexes, depending on which proteins interact with it. The mTOR complex 1 (mTORC1) mainly promotes the transcription of genes involved in cell growth, cell cycle progression and energy metabolism. The mTOR complex 2 (mTORC2) mainly phosphorylates AKT, originating an auto-sustaining positive feedback loop, which results in cell survival and proliferation. Moreover, AKT activation enhances telomerase activity, inhibits apoptosis blocking the release of cytochrome *c* from the mitochondria and inactivating pro-apoptotic factors such as Bad and pro-caspase 9, and regulates modulators of angiogenesis through the activation of nitric oxide synthase [167,168,169,170]. RAS, when interacting with RALGDS, stimulates RAL (RAS-like) GTPases, inducing the activation of phospholipase D1 (PLD) and CDC42/RAC-GAP-RAL binding protein 1 (RALBP1). These, among other pro-survival functions, promote the progression of the cell cycle, inhibiting transcription factors implicated in cell cycle arrest, such as the FORKHEAD transcription factors [131,163]. Another important RAS effector is Tiam 1, which is a Rho family GTPase that regulates the actin cytoskeleton and activates p21 activated protein kinases (PAKs) and c-Jun N-terminal kinase (JNK) [134].

**Figure 8:**
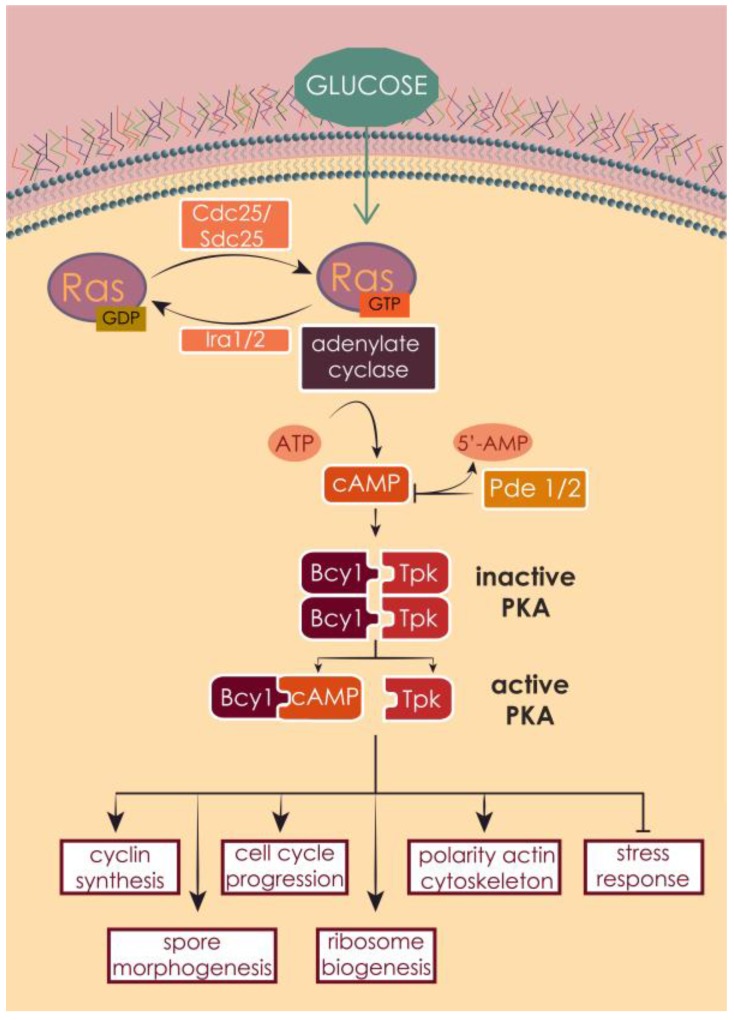
Ras1 and Ras2 are activated upon nutrient availability signaling, glucose in particular. The GEF proteins Cdc25 or Sdc25 catalyze the liberation of GDP and the binding of GTP. Active RAS proteins activate in turn adenylate cyclase, which produces cAMP. This second messenger binds to the inhibitory unit of PKA, releasing the catalytic unit, which phosphorylates multiple downstream targets, leading to the activation of a variety of cellular processes or to the inhibition of transcription factors that control stress response. The pathway can be inactivated by the hydrolysis of cAMP by the phosphodiesterases Pde1 and Pde2, and by the GTPase activity of the GAPs Ira1 and Ira2.

Similarly to human RAS, yeast RAS proteins transduce signals in response to the nutritional conditions of the environment from outside to inside the cell, especially glucose availability [93,171,172] (Figure 8). Contrarily to human RAS and TKRs, the transducer of the growth signal from the outside to the inside of the yeast cell is still not known.

Yeast RAS proteins exert this function mainly in controlling the 3′,5′-cyclic adenosine monophosphate (cAMP) metabolism. cAMP acts as second messenger in the regulation of several fundamental cellular processes, such as protein phosphorylation, accumulation of storage carbohydrates, mitochondrial functions [173,174,175,176,177,178,179,180,181], sporulation [182], sensitivity to heat shock [183] and cell cycle progression [25,184,185,186]. RAS proteins, once activated, interact with adenylate cyclase at the plasma membrane [187], which synthesizes cAMP from guanine nucleotides [27,70,93,172,188,189,190,191,192]. The main target of cAMP is protein kinase A (PKA), which is composed by a catalytic subunit, encoded by the genes *TPK1*, *TPK2* and *TPK3* [193,194], and by a regulatory subunit encoded by *BCY1*. PKA exists in the cell as an inactive heterotetrametric holoenzyme composed by two catalytic and two regulatory subunits [193,194]. cAMP binds to the regulatory unit Bcy1, which relieves its inhibitory effect on the catalytic subunit, allowing the phosphorylation of several downstream targets [171,172,188]. cAMP synthesis is one of several PKA targets, suggesting a strong negative feedback regulation [195,196] and adenylate cyclase itself has been proposed as a PKA target [188]. PKA has a wide variety of substrates, whose activation leads to a dramatic change in the transcriptional program, which helps the cells to adapt to new nutrient conditions, in particular favoring cell growth and proliferation. PKA regulates the metabolism of storage carbohydrates, ribosomal biogenesis, stress response [197,198], polarity of actin cytoskeleton [199], spore morphogenesis [200], cyclins synthesis and subsequent cell cycle progression [25,70,201,202], cell size and growth [171]. 

### 3.6. RAS Effects on Growth, Apoptosis and Autophagy

#### 3.6.1. RAS Effects on Cell Cycle in Human and Yeast

**Figure 9:**
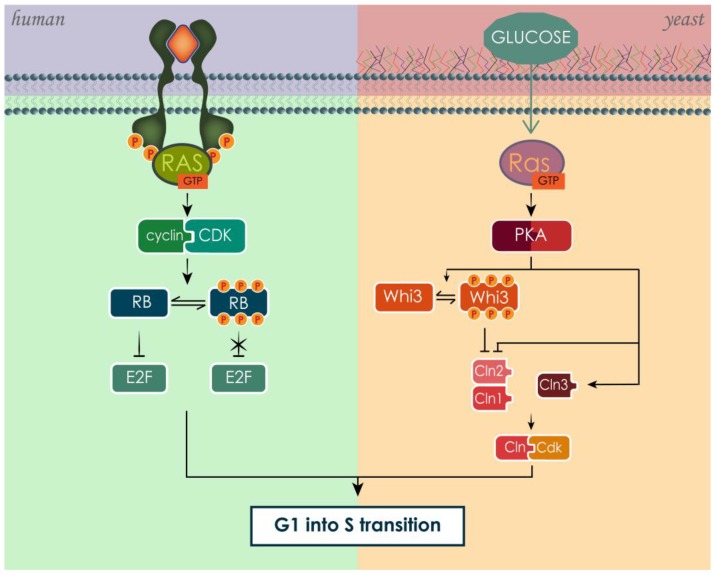
The activation of RAS proteins leads to G1 to S phase progression through the cell cycle in both human and yeast. In both cases, RAS stimulates the formation of functional complexes between cyclins and CDK.

In both human and yeast, RAS is activated in response of the presence of growth and pro-survival signals, specifically growth factors in the case of human RAS [159,161] and glucose in the case of yeast RAS [93,171,172]. Though RAS stimulates growth, meant as an increase in cell number, these effects are not always achieved in the same way in human and yeast due to the different level of organism complexity. RAS stimulates cell cycle progression associated with increase in cell size and protein synthesis [171,197,203,204,205] (Figure 9). However, in the case of human, cell population growth is also promoted by inhibiting apoptosis and stimulating accessory processes such as angiogenesis [163,164,206]. 

The RAS pathway in mammals controls the cell cycle through a variety of different proteins, resulting ultimately in the inactivation of the retinoblastoma (RB) family of pocket proteins [203,207]. RB proteins are found in a hypo-phosphorylated state in resting cells, which confers them the ability to sequester members of the E2F transcription-factor family. These transcription factors, once free from RB inhibition, modulate the transcription of genes involved in the progression of cell cycle from G1 to S phase, especially involved in the regulation of DNA synthesis [205]. The phosphorylation of RB by cyclin-CDK (cyclin-dependent kinase) complexes frees the E2F transcription factors from inhibition. Cyclins and CDKs regulate the progression of the cell cycle by associating in active complexes [204,205]. The connection between RAS-driven pathways and the cell cycle was confirmed by experiments that utilized RAS neutralizing antibodies [208,209,210]. Cells subjected to this type of treatment stopped growth in G1 and presented hypo-phosphorylated RB. On the other hand, expression of oncogenic mutated RAS enabled cells to enter the cell cycle independently from growth factors, progressing through the cell cycle uncontrollably [211,212]. This occurs because the activating mutation of KRAS leads to its constitutive activation, by turning it more resistant to GAPs activity, and consequently, constitutively activating its downstream signaling pathway [79,213]. 

The RAS/cAMP/PKA pathway in yeast regulates not only the cell cycle, but also ribosome production, increase in cell size and mass and growth rate [171,197]. These processes are strictly interdependent. In fact, if on one hand the specific growth rate is determined by the rate of mass accumulation, which in turn depends on nutrient availability, on the other hand cell cycle progression and cell size both depend on specific growth rate and mass accumulation [214,215,216,217,218]. RAS/cAMP/PKA pathway can influence the cell cycle, modulating the expression of cyclins (Clns) [219], whose complexation with cyclin-dependent kinases (Cdks) is necessary to enter in S phase. In particular, RAS suppresses the expression of Cln1 and Cln2, but not of Cln3 [219,220]. Cln3 in this way counteracts the inhibition of the other Clns, mediating their growth-dependent expression [219]. In addition, PKA has been found to directly phosphorylate Whi3, a negative regulator of G1 cyclins, inhibiting its functions and thus promoting the passage into S phase [221].

#### 3.6.2. RAS Effects on Cell Death in Human and Yeast

In addition to regulate cell proliferation, promoting cell cycle progression, RAS pathways control cell survival by modulating apoptosis [222,223,224]. Contrarily to what happens for cell cycle and proliferation, in the case of apoptosis the outcomes of RAS activation are opposite in human and yeast. Indeed, the human RAS pathway is one of the main anti-apoptotic pathways and when up-regulated it can immortalize the cells. On the opposite side, yeast RAS upregulation can lead to programmed cell death through the overexpression of genes that negatively control stress response. 

**Figure 10:**
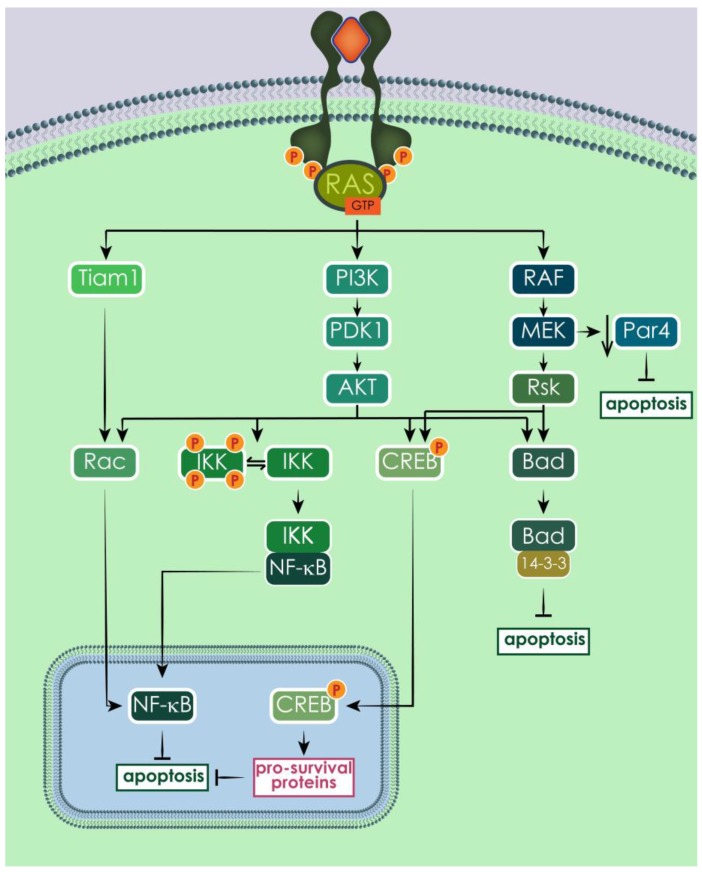
Human RAS proteins protect the cells from apoptosis mainly through the activation of PI3K and the consequent activation of AKT. Among AKT multiple targets there are Rac, IKK, CREB and Bad. Rac can be activated also by Tiam 1, activating in turn NF-κB, an important pro-survival factor. AKT phosphorylates also Bad, promoting the inhibition of the pro-apoptotic factor 14-3-3 and the consequent inhibition of apoptosis, and CREB, promoting the transcription of pro-survival genes. CREB is phosphorylated also by Rsk, activated by RAF/MEK/ERK signaling cascade. In addition, MEK promotes the downregulation of the pro-apoptotic protein Par4, contributing to apoptosis inhibition.

RAS-mediated survival signals promote apoptosis evasion, especially through PI3K pathway. In particular, AKT can phosphorylate Bad, a pro-apoptotic member of the Bcl-2 family, and this phosphorylation causes Bad to bind to 14-3-3 in an inactive complex, instead of sequestering the anti-apoptotic proteins Bcl-2 and Bcl-X_L_ [225]. In addition to AKT, PI3K can activate another important survival factor, NF-κB, through the activation of Rac [226,227,228]. NF-κB is a potent transcription factor that induces the transcription of several anti-apoptotic genes, such as inhibitors of apoptosis proteins (IAPs) [229]. Rac, and consequently NF-κB, can also be activated by RAS directly through Tiam 1, in a PI3K independent manner [230]. A third RAS-mediated pathway to activate NF-κB is the phosphorylation of I-κB kinase (IKK) by AKT [231]. The RAF/MEK/ERK signaling cascade also contributes to the regulation of apoptosis, sometimes converging its signals to the same targets as the PI3K branch, like in the cases of the pro-apoptotic protein Bad [232,233,234,235] and the transcription factor CREB, which induces the expression of pro-survival proteins [233,236]. In addition, RAS has been shown to help escaping apoptosis by downregulating Par-4, a pro-apoptotic transcription repressor, through MEK activity [237], and by inducing p53 degradation, thereby annulling p53-mediated apoptosis induction [238]. Moreover, RAS signal activity through the RAF/MEK/ERK cascade modulates the expression level of several proteins belonging to the Bcl-2 family [239,240] (Figure 10). 

The RAS/cAMP/PKA pathway is a major intracellular player in regulated cell death (RCD) process also in yeast [241,242,243]. It has been observed that increased activation of RAS signaling in yeast cells induces the appearance of typical apoptotic markers, such as phosphatidylserine externalization, increased reactive oxygen species (ROS) accumulation and DNA degradation, among others [244,245]. Three stimuli that lead to RAS/cAMP/PKA hyper-activation and subsequent cell death are osmotin, changes in actin dynamic and ammonium [245,246,247,248]. Osmotin, a protein produced by plants in defense to pathogenic fungi, when in contact with *S. cerevisiae* binds to Pho36, a G-protein-like homologous of the mammalian adiponectin receptor, causing the inappropriate inactivation of RAS signaling, ultimately leading to RCD of the yeast [246]. A different stimulus for RAS-mediated apoptosis in yeast is mediated by actin cytoskeleton. Mutations or addition of drugs can change actin dynamics, causing the formation of F-actin aggregates, which in turn trigger the constitutive activation of Ras2 and apoptosis [245]. In aging yeast cultures, ammonium was found to induce cell dead leading to the shortening of the chronological lifespan, an effect that was mediated through the activation of the RAS/cAMP/PKA pathway [247,248]. The role of mitochondria is fundamental in yeast RCD, this organelle being the main responsible of ROS accumulation when dysfunctional [241]. ROS accumulation is a central event in yeast RCD [249,250,251,252,253] and in both osmotin- and actin-induced RCD, it seems to be the main responsible for cell death, since the addition of antioxidants could suppress the apoptotic phenotype [246,254]. RAS/cAMP/PKA pathway regulates also cell death in acidic environment [255]. Intracellular acidification caused by acetic acid, a known inducer of apoptosis in yeast, leads to RAS/cAMP/PKA activation, which causes consequent cell death [188,250,256]. Supporting the pivotal role of RAS in yeast apoptosis, deletion of *RAS* genes suppresses the apoptotic phenotype of the cells and leads to necrosis instead, while, in an acidic environment, hyper-activation of RAS pathway by constitutively active allele *RAS2^val19^* or by deletion of *PDE2* increases apoptotic cell death [255]. 

#### 3.6.3. RAS Effects on Autophagy in Humans and Yeast

Similarly to what happens for cell cycle and apoptosis, both human and yeast RAS are involved in other cellular processes relevant for cell survival and cancer progression. Indeed, both organisms use RAS proteins, among others, to regulate autophagy. In humans, RAS proteins can promote or inhibit autophagy, depending on the cell context and on the RAS isoform, whereas in yeast RAS activation leads to autophagy repression. The autophagic mechanisms are highly conserved in budding yeast and higher eukaryotes. Autophagy is a catabolic process that targets cellular components, such as damaged cell structures/organelles, long-lived proteins and pathogens for lysosomal degradation [257,258]. Autophagy involves the engulfment of intracellular constituents into double membrane vesicles, termed autophagosomes, which then fuse with lysosomes in mammals or with the vacuole in yeast, where the autophagic contents are degraded [259]. The formation of the autophagosome is controlled by autophagy-related (Atg) proteins, first identified in yeast. More than 30 yeast *ATG* genes have been discovered, and orthologues of many of these genes have been identified and characterized in higher eukaryotes, including humans, suggesting that autophagy is a highly conserved pathway through evolution [260]. At basal levels, autophagy is constitutively active, recycling the cell contents to maintain cellular homeostasis and integrity. Additionally, autophagy can be activated in response to starvation and other conditions of metabolic stress, to provide an alternative source of energy that limits cell death [261]. Autophagy is also implicated in cellular development and differentiation [262], in innate and adaptive immunity [263], as well as in cancer, where its role is highly ambiguous: it may serve as a mechanism of adaptation to stress and consequent avoidance of cell death, or as a route to cell death, by the destruction of the cell itself [264,265,266,267,268,269]. 

In humans, the regulation of the autophagy process converges at the level of mTOR [270], which is one of the downstream effectors of RAS proteins (Figure 7). The best known pathway that regulates mTORC1 is PI3K/AKT and, when activated, it promotes protein synthesis, cell division and metabolism, while autophagy is suppressed. On the other side, RAF/MEK/ERK signaling cascade activated by amino acid starvation can trigger autophagy in human colorectal carcinoma (CRC) [271]. The dual role of RAS activation on autophagy can be observed also for mutated oncogenic RAS proteins and their effects on survival and proliferation. For example, HRAS^G12V^ can either inhibit autophagy by activating PI3K pathway in NIH3T3 [272], or stimulate autophagy through its effects on RAF/MEK/ERK cascade, NOXA or Beclin 1 expression in human ovarian surface epithelial (HOSE) cells, or through RALB (RAS-like protein B) signaling, or the up-regulation of Bnip3 in mouse embryonic fibroblasts (MEF) cells [273,274,275]. In mouse kidney iBMK and human MCF10A mammary epithelial cells, it was reported that overexpression of oncogenic HRAS^G12V^ or KRAS^G12V^ up-regulates autophagy and promotes cell proliferation [276,277,278]. On the other side, there are also reports showing that RAS-driven autophagy is part of a pro-death mechanism. Specifically, infection of ovarian HOSE cells with HRAS^G12V^ induced autophagy and promoted cell death [273]. 

In yeast, the involvement of RAS proteins in autophagy happens mostly through the activity of PKA. Once activated, it phosphorylates Rim15 and Msn2/4, preventing the translocation of these proteins to the nucleus to initiate transcription of the autophagy genes, thereby inhibiting autophagy [279,280]. PKA also inhibits autophagy by direct inhibition of Atg13, which is part of the Atg1 complex (ULK1/2 in mammals), essential for autophagy initiation [281]. TOR pathways are also involved in autophagy regulation in yeast and are highly interconnected with the RAS/cAMP/PKA pathway. TOR and RAS often control overlapping effectors, including the ones involved in autophagy, leading to similar response in the cell, such as inhibition of stress response, aging and cell cycle progression. Importantly, both pathways are activated by the presence of nutrients, glucose in the case of RAS and nitrogen in the case of TOR [171,282,283]. *S. cerevisiae* has two Tor kinases, Tor1 and Tor2, orthologues of human TOR, but only Tor1 gives a significant contribution in the regulation of autophagy [284]. When Tor1 is activated by the presence of nitrogen it represses autophagy by direct inhibition of the Atg1 complex and sequestration of the transcription factors Rim15 and Msn2/4 in the cytoplasm [279,280,281]. Upon starvation induction or treatment with rapamycin, TOR is inhibited and autophagy is induced [285]. In addition to directly inhibit autophagy, TOR also exerts its functions through its main downstream effector Sch9, which is directly phosphorylated and activated by the TORC1 complex [286]. The inactivation of Sch9p and PKA is sufficient to trigger autophagy, suggesting that these kinases are cooperatively involved in negative regulation of this process [287]. 

## 4. Involvement of RAS Proteins in Cancer: Yeast as a Model Organism

The role of human RAS proteins in carcinogenesis is well established. Approximately 30% of all human cancers harbors an activating point mutation in *RAS*, with pancreas (60–90%), colon (30–50%) and lung (20–30%) cancers displaying the highest frequency [79,163,288]. KRAS mutations are common in pancreatic, colorectal, endometrial, biliary tract, lung and cervical cancers. NRAS mutations are more prevalent in myeloid leukemia and HRAS mutations predominate in bladder cancer [79,288,289]. RAS point mutations are found at the highest frequency at codons 12, 13 and 61. 

The uncontrolled cell growth typical of tumors with mutated *RAS* is due to the hyper-activation of RAS proteins. Indeed, mutated RAS are more resistant to GAPs activity. Without being efficiently affected by GAPs, their endogenous GTP hydrolysis is too low and, in this way, RAS proteins are locked in a permanent active state, which increases RAS activity and its downstream signaling [79]. Hyper-activation of RAS signaling can be caused as well by mutations in genes encoding proteins that interact with RAS [290,291]. Another factor that causes hyper-activation of RAS signaling is the overexpression of EGFR [292]. Mutations or amplification of downstream RAS effectors have also been implicated in human cancer development [293,294,295].

**Figure 11:**
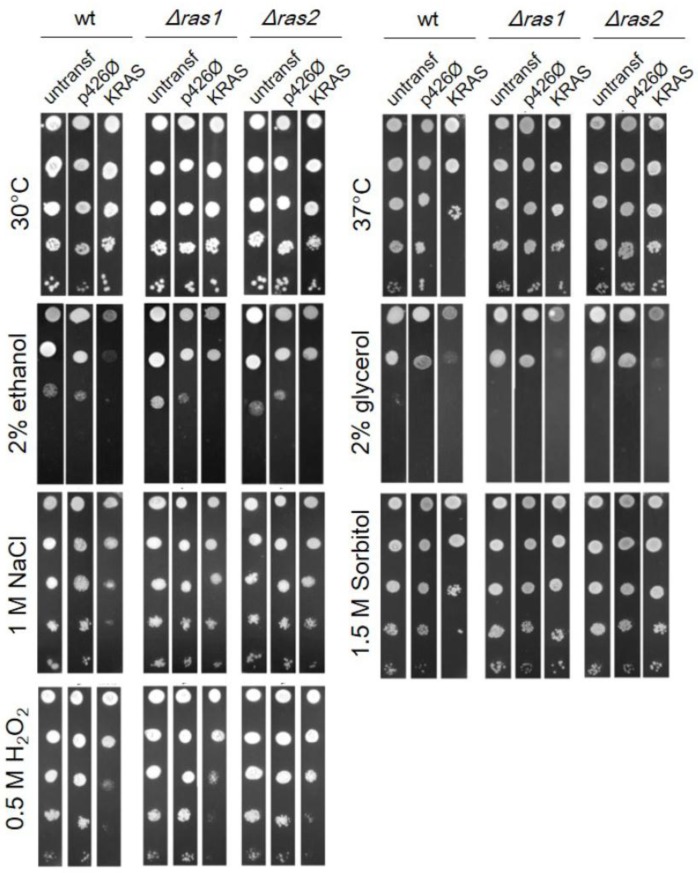
Serial drop test of BY4741 strain expressing human KRAS. *S. cerevisiae* BY4741 wt, *Δras1* and *Δras2* were subjected to different stress conditions (stress stimulus described on the side of each box). The direction of the serial dilutions, from 10^−1^ to 10^−5^, proceeds from top to bottom. The image is representative of one of three independent experiments.

Besides the aforementioned contribution of *S. cerevisiae* to clarifying several aspects of mammalian RAS upstream regulation, further evidence has sustained the rationale behind the use of this model organism to study RAS functioning. Mammalian RAS proteins can indeed act as a direct complement for yeast-deficient RAS and vice versa. In the middle of the 1980s, several studies showed that activated human HRAS could suppress the lethality of simultaneous deletion of *RAS1* and *RAS2* in yeast, because it shows the same ability to activate yeast adenylate cyclase as yeast Ras1 or Ras2 [23,24,26,68,296]. The functional complementation of *RAS2* deletion in yeast by human *HRAS* was later proved also for different *Δras2*-induced phenotypes, such as temperature-sensitive growth and temperature-dependent depolarization of the actin cytoskeleton [199]. Conversely, it was shown that a yeast-mammalian hybrid gene expressed in mouse cells had the ability to induce morphological changes extremely similar to those induced by mammalian oncogenic HRAS [23]. In addition, a mutant variant of yeast RAS protein that resembles oncogenic HRAS was also identified, Ras^val19^, which was capable to differentially activate adenylate cyclase [26,296]. KRAS, in opposition to HRAS, is not able to complement the double deletion of *RAS1* and *RAS2*, resulting in a non-viable yeast [23,24]. Results from our group [297] showed that the expression of human *KRAS* in yeast expressing its own *RAS1* and *RAS2* genes (wt) caused a decrease in the strain resistance to high non-permissive temperature, osmotic stress and oxidative stress (Figure 11). Moreover, both wt and *∆ras1* or *∆ras2* yeast strains expressing human *KRAS* were less able to grow on non-fermentable carbon sources like ethanol and glycerol (Figure 11). Exogenous human RAS protein, in addition to the yeast endogenous Ras1 and Ras2, increased sensitivity to stress in wild type, but this phenotype was less pronounced or absent in *RAS* deletion strains. So KRAS seems to be able to activate yeast RAS downstream cascades, even if it cannot complement the growth defect caused by the loss of both *RAS* genes [297]. This strongly suggests that human KRAS, once inside the yeast cell, has roles that are probably very close to the ones of the endogenous RAS proteins.

### 4.1. S. cerevisiae as a Model for Studying KRAS-Induced Autophagy in Colorectal Cancer

Recently, our group addressed the role of KRAS proteins in autophagy modulation using yeast as a model [17]. As mentioned above, autophagy is a highly conserved metabolic process that cells use to recycle their components and maintain cellular homeostasis [298]. Importantly, autophagy is also conserved in yeast [260]. In cancer, autophagy is involved on a double front; on one side, autophagy can weaken the cells by degrading their components, but on the other side, the recycling of peptides makes the cells less sensitive to nutrient depletion and therefore to death. This pro-survival role has been evidenced by studies showing that autophagy is implicated in the resistance of tumors to chemotherapy [299]. The RAS pathway is one of the major regulators of autophagy [272,273,275,276,277,278]. Work using a yeast strain lacking *RAS2* and transformed with either *KRAS^wt^* or its most common mutated alleles (*KRAS^G13D^*, *KRAS^G12D^*, *KRAS^G12V^*) in colorectal carcinoma made it possible to uncover that the activating KRAS mutations, unlike wild-type KRAS, increase the level of the yeast autophagy reporter Atg8 [17]. Further studies with CRC cell lines confirmed that *KRAS* mutated alleles increased autophagy, and showed that this increase was associated with increased survival under starvation conditions. KRAS-induced autophagy was mediated through upregulation of the RAS/RAF/MEK/ERK pathway and downregulation of the PI3K/AKT pathway, known to activate the autophagy inhibitor mTOR. The humanized yeast in this case helped to determine the role of mutated and wild type KRAS in the autophagic process. This study reinforces that human RAS proteins are functional in yeast and that, as in mammalian cells, they can activate the autophagic machinery, further supporting yeast as an excellent model to study human RAS and its involvement in tumorigenesis through the modulation of autophagy.

### 4.2. S. cerevisiae as a Model for Studying KRAS/gal-3 Interaction in Colorectal Cancer

**Figure 12:**
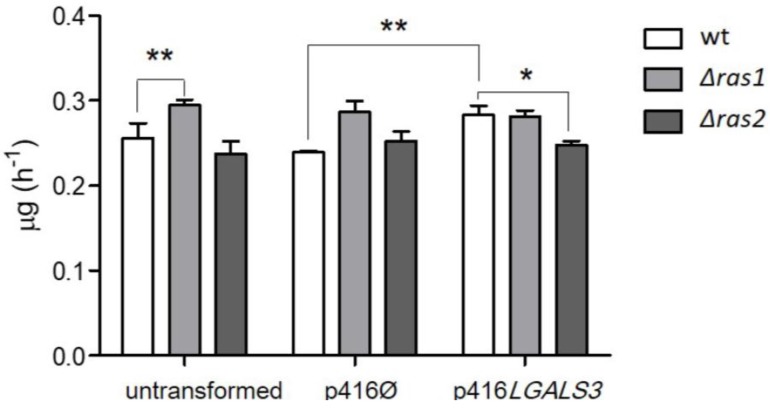
*S. cerevisiae* wt, *Δras1* and *Δras2* transformed with p416*LGALS3* were grown on glucose for 28 h from O.D._600_ 0.05 up to stationary phase. The untransformed yeasts and the yeasts transformed with the empty plasmid (p416 Ø) were used as controls. Specific growth rates were estimated from log phase. Graphs show the average specific growth rate ± SD of three independent experiments. Statistical significant differences are shown: * (*p*-value ≤ 0.05) and ** (*p*-value ≤ 0.01).

In addition to showing that human RAS can interact with endogenous yeast proteins, as discussed above for autophagy, we recently disclosed that, reciprocally, yeast RAS activity can be influenced by heterologously expressed human proteins, namely galectin-3 [297]. KRAS has been found to interact specifically with gal-3 in the cytoplasm [137,151,300]. It has been hypothesized that gal-3 may render KRAS less sensitive to GAPs and therefore maintain KRAS in a constitutive active state, increasing the pro-growth signaling transmitted by KRAS. Indeed, it has been noticed that the interaction between gal-3 and KRAS enhances PI3K activity and Raf-1 activation [151]. Gal-3 enhances KRAS activity acting as a scaffold protein, maintaining it in a proper orientation, relevant for its activity regulation [301], and in organized nanoclusters on the cell membrane, facilitating its function [137,152,302]. In addition, it seems that the expression of gal-3 increases not only the activation, but also the expression of KRAS [303]. Since KRAS promotes cell proliferation and inhibition of apoptosis and gal-3 appears to enhance KRAS activity through different mechanisms, it is conceivable that this interaction enhances cancer progression. It has been shown that gal-3 interaction with KRAS potentiates thyroid cancer progression, increasing KRAS signaling and thus proliferation [303]. In addition, gal-3 has been found overexpressed in pancreatic cancer, where it interacts with KRAS-GTP, influencing its active status and its membrane localization. Alterations in gal-3 level and consequent variation in RAS downstream signaling cascades strongly modulate the cancer phenotype. Downregulation of gal-3 decreases growth, invasiveness, anchorage independent growth and tumor growth in an in vivo orthotopic model, whereas gal-3 upregulation stimulates growth proliferation [304]. A colon cancer cell line was found to express gal-3 at high levels, and this expression correlates with the migration ability of cancer cells [305]. Further highlighting the relationship between gal-3 and the RAS pathway in colon cancer, a correlation between high levels of gal-3, RAF and ERK was found tissue samples [305]. Finally, gal-3 seems to be the adaptor for the interaction between KRAS and α_ν_β_3_ integrin, which causes tumor aggressiveness [306,307]. This further proves the fundamental role of gal-3 in mediating KRAS function and its ability to ablate KRAS-mediated pro-survival signals, when downregulated. To summarize, it has been discovered that an increased expression or availability of cytoplasmic gal-3 could confer to the cells that same tumorigenic properties—namely uncontrolled growth and downregulated apoptosis—as the mutation of an oncogene, in this case *KRAS.* This fact emphasizes the need to better understand the interaction between these two proteins. For all these reasons, we expressed human gal-3 in yeast and measured some basic phenotypes, such as specific growth rate. Our aim was to build a humanized yeast that could be used as a model to study the interaction between KRAS and gal-3. We observed that the presence of endogenous yeast RAS proteins greatly affected the outcomes of gal-3 expression in yeast. In particular, an increase in the growth rate was observed only in the wild type yeast and not in the two *RAS* deletion mutants when gal-3 was expressed [297]. In human cells, gal-3 interacts with KRAS, stabilizing its active GTP-bound form [151,300,303,304] and its positioning on the inner side of the plasma membrane, the proper location for RAS signaling [137,151,302]. This observation suggests that yeast RAS proteins might interact as well with gal-3. This RAS/gal-3 interaction could therefore have similar effects in yeast and in human cells, namely the stabilization and activation of RAS proteins by gal-3, enhancing RAS signaling, and in this way increasing the growth rate (Figure 12) [297]. In support of this hypothesis is the fact that several other mammalian RAS partners could successfully interact with yeast RAS proteins, like the above-mentioned mammalian GTP activating protein NF1 [77,308] and proteins involved in the autophagic process [17].

## 5. Final Remarks 

This review highlights the main similarities and differences between human and yeast RAS proteins and pathways. It becomes clear that RAS proteins from the two organisms share similar features, like the sequence, structure, mechanism of activation and the cellular outcomes they cause. This can still occur, though the intracellular partners and the pathways that RAS proteins activate in human and yeast differ. These differences are yet not enough to prevent RAS regulators and effectors to be functionally interchangeable in the two organisms. The main contributions of *S. cerevisiae* in clarifying different aspects of mammalian RAS regulation, such as the PTMs necessary for membrane anchorage [71,72], GAPs [79,80] and GEFs [75,76] promoting GTP/GDP binding, as well as the role of RAS in regulation of cell cycle, growth, cell death and autophagy, are also stressed. Yeast has been specifically used to analyze the role of human KRAS in autophagy, a cancer-related process, and was established as a model to study KRAS-induced autophagy. 

Here, we wanted to evidence that yeast is a useful model in the study of human RAS proteins because of the functional conservation of RAS proteins roles. Though increasing advanced resources are becoming more available to study higher eukaryotic organisms, the “simple” yeast eukaryotic model together with novel and expedite tools, still seems promising to genetically analyze the molecular mechanisms at the core of human RAS signal transduction pathways and their involvement in tumorigenesis. 

## Appendix A

In the text, genes and proteins have been written accordingly to the standard nomenclature of the specific organism. In the case of *S. cerevisiae*, wild-type genes were presented as all italicized capital letters and specific yeast proteins as non-italicized, with only the first letter in upper case. Human genes and proteins were written all in capital letters, with the difference that gene names were italicized, whereas protein names were not [309,310]. When RAS proteins were addressed in general, without specifying the exact protein, either in human or yeast, they were written in upper case, non-italicized.
